# Characteristics of Patients Experiencing Rehospitalization or Death After Hospital Discharge in a Nationwide Outbreak of E-cigarette, or Vaping, Product Use–Associated Lung Injury — United States, 2019

**DOI:** 10.15585/mmwr.mm685152e1

**Published:** 2020-01-03

**Authors:** Christina A. Mikosz, Melissa Danielson, Kayla N. Anderson, Lori A. Pollack, Dustin W. Currie, Rashid Njai, Mary E. Evans, Alyson B. Goodman, Evelyn Twentyman, Jennifer L. Wiltz, Dale A. Rose, Vikram Krishnasamy, Brian A. King, Christopher M. Jones, Peter Briss, Matthew Lozier, Sascha Ellington, Lung Injury Response Epidemiology/Surveillance Task Force, Lung Injury Response Epidemiology/Surveillance Task Force, Adebola Adebayo, Sukhshant Atti, Amy Board, Chelsea Austin, Gyan Chandra, Kelsey Coy, Alissa Cyrus, Geroncio Fajardo, Sonal Goyal, Sierra Graves, Janet Hamilton, Donald Hayes, Denise Hughes, Mia Israel, Jean Ko, Zheng Li, Ruth Lynfield, Suzanne Newton, Sharyn Parks, Mary Pomeroy, Katherine Roguski, Phillip Salvatore, Caroline Schrodt, Stephen Soroka, Kimberly Thomas, Bailey Wallace, Megan Wallace

**Affiliations:** ^1^National Center for Injury Prevention and Control, CDC; ^2^National Center on Birth Defects and Developmental Disabilities, CDC; ^3^National Center for Chronic Disease Prevention and Health Promotion, CDC; ^4^Center for Global Health, CDC; ^5^Epidemic Intelligence Service, CDC; ^6^Office of the Director, Deputy Director for Noninfectious Diseases, CDC; ^7^National Center for Emerging and Zoonotic Infectious Diseases, CDC.; National Center for Injury Prevention and Control, CDC; Agency For Toxic Substances and Disease Registry, CDC; National Center for Injury Prevention and Control, CDC; National Center for Environmental Health, CDC; National Center for Chronic Disease Prevention and Health Promotion, CDC; National Center for Chronic Disease Prevention and Health Promotion, CDC; Office of Minority Health and Health Equity, CDC; National Center for Emerging and Zoonotic Infectious Diseases, CDC; National Center for Chronic Disease Prevention and Health Promotion, CDC; National Center on Birth Defects and Developmental Disabilities, CDC; Council of State and Territorial Epidemiologists; National Center for Chronic Disease Prevention and Health Promotion, CDC; National Center for Immunization and Respiratory Diseases, CDC; Council of State and Territorial Epidemiologists; National Center for Chronic Disease Prevention and Health Promotion, CDC; Agency For Toxic Substances and Disease Registry, CDC; Minnesota Department of Health; National Center on Birth Defects and Developmental Disabilities, CDC; National Center for Chronic Disease Prevention and Health Promotion, CDC; National Center for Emerging and Zoonotic Infectious Diseases, CDC; National Center for Emerging and Zoonotic Infectious Diseases, CDC; National Center for Injury Prevention and Control, CDC; National Center for Emerging and Zoonotic Infectious Diseases, CDC; National Center for Emerging and Zoonotic Infectious Diseases, CDC; Center for Surveillance, Epidemiology, and Laboratory Services, CDC; National Center on Birth Defects and Developmental Disabilities, CDC; National Center for Immunization and Respiratory Diseases, CDC.

CDC, the Food and Drug Administration (FDA), state and local health departments, and public health and clinical stakeholders continue to investigate a nationwide outbreak of e-cigarette, or vaping, product use–associated lung injury (EVALI) (*1*–*4*). Characterizing EVALI patients who experience rehospitalization or death after hospital discharge might identify risk factors for higher morbidity and mortality. CDC analyzed national data on EVALI patients to determine the prevalence of rehospitalization and death after discharge and to identify characteristics associated with EVALI patients who require rehospitalization and those who die after discharge, compared with other EVALI patients. As of December 10, 2019, a total of 2,409 EVALI cases requiring hospitalization have been reported to CDC, as have 52 deaths. Among the 1,139 EVALI patients discharged on or before October 31, 2019, 31 (2.7%) were rehospitalized after discharge, with a median of 4 days (interquartile range [IQR] = 2–20 days) between discharge and rehospitalization; seven deaths (13.5% of EVALI deaths) occurred after discharge, with a median of 3 days (IQR = 2–13 days) between discharge and death. Characteristics of EVALI patients who were rehospitalized or died after hospital discharge suggest that chronic medical conditions, including cardiac disease, chronic pulmonary disease (e.g., chronic obstructive pulmonary disease [COPD] and obstructive sleep apnea), and diabetes, are risk factors leading to higher morbidity and mortality among some EVALI patients. For example, 70.6% of patients who were rehospitalized and 83.3% of those who died had one or more chronic conditions, compared with 25.6% of those who were neither rehospitalized nor died. In addition, EVALI patients who were rehospitalized or died after discharge were older: the median ages of patients who died, were rehospitalized, and who neither died nor were rehospitalized were 54, 27, and 23 years, respectively. EVALI patient follow-up optimally within 48 hours after hospital discharge might minimize risk for rehospitalization and death, especially among patients with chronic conditions. In addition, interventions for EVALI patients, including intensive hospital discharge planning and optimized case management, might minimize risks for morbidity and mortality after a hospital discharge (*5*).

CDC partnered with state health departments and the Council of State and Territorial Epidemiologists Vaping Associated Pulmonary Illness Task Force to develop and disseminate EVALI surveillance case definitions* and data collection tools^†^ beginning in August 2019. States and jurisdictions voluntarily report data on confirmed and probable hospitalized or deceased EVALI patients to CDC weekly. States might also include available data from medical record abstractions and interviews of patients, or proxies (e.g., spouses or parents) if patients were too ill or had died.

This report compares clinical characteristics of EVALI patients who were rehospitalized or died after hospital discharge with those of patients who were neither rehospitalized nor died after hospital discharge, among cases reported to CDC by December 10, 2019. Rehospitalized patients were defined as those who had a second hospitalization, regardless of reason for admission, that occurred one or more days after the date of discharge from the first hospitalization. A death after hospital discharge was defined as death, regardless of reason for death, that occurred one or more days after the date of last hospital discharge. Rehospitalized patients and those who died after discharge were compared separately with hospitalized EVALI patients who met the following criteria: 1) an initial hospital discharge date on or before October 31, 2019, to allow time for the two outcomes of interest to potentially occur; 2) no reports of rehospitalization nor death as of December 10, 2019; and 3) available data for at least one variable in all of the following categories: medical history, EVALI symptoms reported, and clinical course of EVALI illness. Percentages and distributions of categorical and continuous indicators were compared using Fisher’s exact tests and Kruskal-Wallis tests, respectively; p-values <0.05 were considered statistically significant for pair-wise comparisons between 1) the comparison group and patients who were rehospitalized or 2) the comparison group and those who died after discharge. To assess the impact of multiple comorbidities on rehospitalization or death after discharge among EVALI patients, the additive effect of several specific chronic conditions was studied; chronic conditions included for this comorbidity analysis were cardiac disease; asthma; obstructive sleep apnea; COPD; other respiratory conditions not including asthma, obstructive sleep apnea, or COPD (e.g., interstitial lung disease); and diabetes. The IQR was included where median values were reported. All analyses were conducted using SAS software (version 9.4; SAS Institute).

As of December 10, 2019, a total of 2,409 EVALI cases requiring hospitalization have been reported to CDC, as have 52 deaths. Among the 1,139 EVALI patients discharged on or before October 31, 2019, 31 (2.7%) were rehospitalized after discharge without subsequent report of death. An additional seven deaths (13.5% of EVALI deaths) occurred after hospital discharge. The comparison group included 768 EVALI patients who met the inclusion criteria. The age distributions differed among EVALI patients who were rehospitalized, who died after discharge, and who were neither rehospitalized nor died (Table 1). The median ages of patients who died, were rehospitalized, and who neither died nor were rehospitalized were 54, 27, and 23 years, respectively. Among deaths after discharge, five (71.4%) occurred among females, although females accounted for 33.6% of comparison cases.

**TABLE 1:** Demographic and medical history characteristics of e-cigarette, or vaping, product use–associated lung injury (EVALI) patients, by rehospitalization, death after discharge, and no rehospitalization nor death after discharge — United States, 2019*

Characteristic	Rehospitalization (N = 31)		P-value^§^	Death after discharge (N = 7)		P-value^¶^	No rehospitalization nor death^†^ (N = 768)	
	No.	No. (%) or median (IQR)		No.	No. (%) or median (IQR)		No.	No. (%) or median (IQR)
Age, median (IQR)	**31**	**27 (17–39)**	**0.35**	**7**	**54 (34–75)**	**<0.001**	**766**	**23 (18–32)**
**Age group (yrs)**								
13–17	31	8 (25.8%)	0.01	7	0 (0.0%)	<0.001	766	128 (16.7%)
18–24		4 (12.9%)			0 (0.0%)			305 (39.8%)
25–50		17 (54.8%)			2 (28.6%)			290 (37.9%)
≥51		2 (6.5%)			5 (71.4%)			43 (5.6%)
**Gender**								
Male	31	18 (58.1%)	0.36	7	2 (28.6%)	0.06	766	508 (66.3%)
Female		13 (41.9%)			5 (71.4%)			257 (33.6%)
Other		0 (0.0%)			0 (0.0%)			1 (0.1%)
**Medical history**								
Any cardiac disease	16	4 (25.0%)	0.07	6	5 (83.3%)	<0.001	591	59 (10.0%)
Any chronic respiratory disease	22	10 (45.5%)	0.09	5	2 (40.0%)	0.62	681	187 (27.5%)
Asthma	16	3 (18.8%)	0.74	5	0 (0.0%)	>0.99	599	99 (16.5%)
Obstructive sleep apnea	16	3 (18.8%)	0.002	5	2 (40.0%)	0.002	599	8 (1.3%)
COPD	16	2 (12.5%)	0.12	5	2 (40.0%)	0.01	599	21 (3.5%)
Diabetes mellitus	16	3 (18.8%)	0.009	5	1 (20.0%)	0.13	599	15 (2.5%)
Any mental, emotional, or behavioral disorder	19	13 (68.4%)	0.10	5	4 (80.0%)	0.20	645	310 (48.1%)
Anxiety	17	10 (58.8%)	0.13	5	3 (60.0%)	0.38	558	214 (38.4%)
Depression	16	5 (31.3%)	0.80	5	3 (60.0%)	0.37	553	204 (36.9%)
ADHD	16	2 (12.5%)	0.19	5	0 (0.0%)	>0.99	599	29 (4.8%)
Chronic conditions**								
Presence of ≥1 chronic condition	17	12 (70.6%)	<0.001	6	5 (83.3%)	0.006	665	170 (25.6%)
Presence of ≥2 chronic conditions		3 (17.6%)	0.03		3 (50.0%)	0.001		25 (3.8%)
No. of chronic conditions^†^ (median [IQR])		1 (0–1)	<0.001		1.5 (1–3)	<0.001		0 (0–1)

**Abbreviations:** ADHD = attention deficit hyperactivity disorder; COPD = chronic obstructive pulmonary disease; IQR = interquartile range.

* For cases reported by December 10, 2019.

^†^ Includes hospitalized EVALI patients who met the following criteria: 1) an initial hospital discharge date on or before October 31, 2019; 2) no reports of rehospitalization nor death as of December 10, 2019; and 3) available data for at least one variable in all of the following categories: medical history, EVALI symptoms reported, and clinical course of EVALI illness.

^§^ Comparing EVALI patients who were rehospitalized to those who were not rehospitalized nor died. Fisher’s exact tests were used to compare categorical variables, and Kruskal-Wallis tests were used to compare continuous variables.

^¶^ Comparing EVALI patients who died after discharge to those who were not rehospitalized nor died. Fisher’s exact tests were used to compare categorical variables, and Kruskal-Wallis tests were used to compare continuous variables.

** Chronic conditions included here are cardiac disease; asthma; obstructive sleep apnea (OSA); COPD; other respiratory conditions not including asthma, OSA, or COPD; and diabetes mellitus. Examples of “other respiratory conditions” observed among EVALI patients included interstitial lung disease, pulmonary hypertension, and hypersensitivity pneumonitis.

EVALI patients who were rehospitalized or died after hospital discharge had more chronic medical conditions. For example, 70.6% and 17.6% of patients who were rehospitalized had at least one or at least two chronic conditions, respectively, and 83.3% and 50.0% of those who died had at least one or at least two chronic conditions, respectively, compared with 25.6% and 3.8%, respectively, of those who were neither rehospitalized nor died (p<0.05) (Table 1).

Neither symptoms reported when initially seeking medical care nor the location of this initial care were associated with rehospitalization or death after discharge (Table 2). All patients who died after hospital discharge had been admitted to an intensive care unit during their previous hospitalization (p = 0.006), compared with 41.9% of the comparison group and 47.4% of the surviving rehospitalized patients (Table 3). Respiratory failure necessitating intubation and mechanical ventilation during initial hospitalization was more common among patients who died (100%) than among patients who were neither rehospitalized nor died (15.6%) (p = 0.03). No significant difference among the three groups with respect to receipt of corticosteroid therapy or antibiotic therapy during initial hospitalization was observed. Duration of initial hospitalization did not differ among the three groups. Among rehospitalized patients, a median of 4 days (IQR = 2–20) elapsed between discharge from the first hospitalization and rehospitalization. Among patients who died after discharge, a median of 3 days (IQR = 2–13) elapsed between hospital discharge and death.

**TABLE 2:** Clinical characteristics upon first reported clinical encounter of e-cigarette, or vaping, product use–associated lung injury (EVALI) patients, by rehospitalization, death after discharge, and no rehospitalization nor death after discharge — United States, 2019*

Characteristic	Rehospitalization (N = 31)			Death after discharge (N = 7)			No rehospitalization nor death^†^ (N = 768)	
	No.	No. (%) or median (IQR)	P-value^§^	No.	No. (%) or median (IQR)	P-value^¶^	No.	No. (%) or median (IQR)
**Symptoms at first reported clinical encounter**								
Any respiratory**	25	25 (100%)	0.62	7	7 (100%)	>0.99	760	726 (95.5%)
Any gastrointestinal^††^	24	19 (79.2%)	0.79	6	4 (66.7%)	0.31	732	598 (81.7%)
Any constitutional^§§^	25	21 (84.0%)	0.14	7	5 (71.4%)	0.10	743	684 (92.1%)
Days between date of symptom onset and first clinical encounter	23	6 (1–15)	0.35	7	3 (1–5)	0.09	679	5 (3–8)
**Location of first reported clinical encounter**								
Hospital^¶¶^	31	25 (80.6%)	0.76	7	5 (71.4%)	0.84	762	554 (72.7%)
Emergency department only***		3 (9.7%)			1 (14.3%)			117 (15.4%)
Outpatient/Urgent care		3 (9.7%)			1 (14.3%)			91 (11.9%)

**Abbreviation**: IQR = interquartile range.

* For cases reported by December 10, 2019.

^†^ Includes hospitalized EVALI patients who met the following criteria: 1) an initial hospital discharge date on or before October 31, 2019; 2) no reports of rehospitalization nor death as of December 10, 2019; and 3) available data for at least one variable in all of the following categories: medical history, EVALI symptoms reported, and clinical course of EVALI illness.

^§^ Comparing EVALI patients who were rehospitalized to those who were not rehospitalized nor died. Fisher’s exact tests were used to compare categorical variables, and Kruskal-Wallis tests were used to compare continuous variables.

^¶^ Comparing EVALI patients who died after discharge to those who were not rehospitalized nor died. Fisher’s exact tests were used to compare categorical variables, and Kruskal-Wallis tests were used to compare continuous variables.

** Common examples include: cough, shortness of breath, chest pain, and difficulty breathing.

^††^ Common examples include: diarrhea, nausea, vomiting, and abdominal pain.

^§§^ Common examples include: fever, chills, malaise, fatigue, headache, and body aches.

^¶¶^ Includes hospitalizations that occurred directly from the emergency department.

*** Does not include emergency department encounters resulting in hospitalization.

**TABLE 3:** Clinical course during initial hospitalization of e-cigarette, or vaping, product use–associated lung injury (EVALI) patients, by rehospitalization, death after discharge, and no rehospitalization nor death after discharge — United States, 2019*

Characteristic	Rehospitalization (N = 31)			Death after discharge (N = 7)			No rehospitalization nor death^†^ (N = 768)	
	No.	No. (%) or median (IQR)	P-value^§^	No.	No. (%) or median (IQR)	P-value^¶^	No.	No. (%) or median (IQR)
Admission to intensive care unit	19	9 (47.4%)	0.65	6	6 (100%)	0.006	677	284 (41.9%)
Respiratory failure necessitating intubation and mechanical ventilation	11	4 (36.4%)	0.08	2	2 (100%)	0.03	347	54 (15.6%)
Extracorporeal membrane oxygenation	15	0 (0.0%)	>0.99	5	1 (20.0%)	0.05	459	4 (0.9%)
**Corticosteroids**	19	17 (89.5%)	>0.99	5	5 (100%)	>0.99	629	555 (88.2%)
Days after initial hospital admission that steroid treatment was initiated	6	1.5 (0–3)	0.40	1	9	N/A	200	1 (0–3)
Duration of steroid treatment (days)	3	20 (5–30)	0.38	1	18	N/A	97	10 (5–17)
**Antibiotics received**	15	15 (100%)	>0.99	4	4 (100%)	>0.99	518	507 (97.9%)
**Imaging**								
**CT performed**	16	16 (100%)	0.38	6	6 (100%)	>0.99	547	498 (91.0%)
Any infiltrates or opacities on CT	11	11 (100%)	>0.99	2	2 (100%)	>0.99	254	253 (99.6%)
Bilateral findings on CT	10	10 (100%)	>0.99	2	2 (100%)	>0.99	254	244 (96.1%)
**X-ray performed**	16	16 (100%)	>0.99	6	6 (100%)	>0.99	538	522 (97.0%)
Any infiltrates or opacities on x-ray	7	5 (71.4%)	0.13	2	2 (100%)	>0.99	249	227 (91.2%)
Bilateral findings on x-ray	10	6 (60.0%)	0.23	2	2 (100%)	>0.99	262	206 (78.6%)
**CT, x-ray, or both performed**	17	17 (100%)	>0.99	6	6 (100%)	>0.99	578	578 (100%)
Any infiltrates or opacities	11	11 (100%)	>0.99	2	2 (100%)	>0.99	307	307 (100%)
Bilateral findings on CT, x-ray, or both	11	10 (90.9%)	0.51	2	2 (100%)	>0.99	308	289 (93.8%)
**Duration of hospitalization (days)**								
First admission	31	4 (2–8)	0.11	7	9 (2–23)	0.33	762	5 (3–8)
Second admission	27	4 (2–8)	N/A	N/A	N/A	N/A	N/A	N/A
**Days between discharge from first hospitalization and admission for second hospitalization**	31	4 (2–20)	N/A	1	1	N/A	N/A	N/A
**Days between discharge from first hospitalization and death**	N/A	N/A	N/A	7	3 (2–13)	N/A	N/A	N/A

**Abbreviations:** CT = computed tomography; IQR = interquartile range; N/A = not applicable.

* For cases reported by December 10, 2019.

^†^ Includes hospitalized EVALI patients who met the following criteria: 1) an initial hospital discharge date on or before October 31, 2019; 2) no reports of rehospitalization nor death as of December 10, 2019; and 3) available data for at least one variable in all of the following categories: medical history, EVALI symptoms reported, and clinical course of EVALI illness.

^§^ Comparing EVALI patients who were rehospitalized to those who were not rehospitalized nor died. Fisher’s exact tests were used to compare categorical variables, and Kruskal-Wallis tests were used to compare continuous variables.

^¶^ Comparing EVALI patients who died after discharge to those who were not rehospitalized nor died. Fisher’s exact tests were used to compare categorical variables, and Kruskal-Wallis tests were used to compare continuous variables.

## Discussion

As of December 10, 2019, 2.7% of EVALI patients reported to CDC have required rehospitalization, and approximately one in seven deaths among EVALI patients has occurred after discharge. Compared with other hospitalized EVALI patients, the prevalence of one or more chronic conditions was higher among those who required rehospitalization and those who died after discharge. EVALI patients who died also were more likely to have been admitted to an intensive care unit, experienced respiratory failure necessitating intubation and mechanical ventilation, and were significantly older. EVALI patients with chronic comorbidities and these initial hospitalization characteristics might require a higher threshold for hospital discharge and focused efforts during discharge planning and transition to the outpatient setting, such as intensive case management and rapid follow-up (*5*).

At least one quarter of rehospitalizations occurred within 2 days of initial discharge, which suggests that ensuring clinical stability before discharge as well as postdischarge follow-up optimally within 48 hours might minimize risk for rehospitalization and death, especially among patients with chronic conditions (*5*). A higher frequency of rehospitalizations among EVALI patients after a longer interval has been reported elsewhere (*6*); differences observed in the current study might reflect its larger study population and wider geographic distribution of EVALI cases. Concurrent with this report, CDC is publishing additional clinical guidance for discharge planning for EVALI patients (*5*).

The findings in this report are subject to at least seven limitations. First, the limited proportion of reported cases with detailed clinical data might limit generalizability. Second, the small number of rehospitalizations and deaths after discharge limit the ability to identify significant differences and assess confounding factors. Third, EVALI patients in the comparison group might not fully represent a cohort at lower risk; some patients might still be rehospitalized or die. However, limiting comparison cases to those patients discharged on or before October 31, 2019, was intended to mitigate this effect. Fourth, reported data do not include the reason for rehospitalization or death after hospital discharge of EVALI patients; rehospitalization or death was possibly not related to EVALI, especially among patients with multiple comorbidities. Fifth, use of e-cigarette, or vaping, products, as well as compliance with recommended postdischarge treatment, was not assessed. Sixth, available data might represent an underestimation of rehospitalized EVALI patients. These data might not be as rigorously reported as those concerning patients initially seeking care, there might have been variability in how states defined rehospitalization, or both. Finally, data on insurance status were not collected, so the relationship between EVALI outcomes and insurance status, prescription medication coverage, and access to care in the inpatient and outpatient settings could not be assessed.

Among EVALI patients, careful and comprehensive discharge planning, ensuring clinical stability before discharge, follow-up optimally within 48 hours after hospital discharge, and enhanced efforts to coordinate care and address comorbidities might minimize risk for rehospitalizations or death after discharge (*5*). The latest national and state data from patient reports and product sample testing suggest tetrahydrocannabinol-containing e-cigarette, or vaping, products, particularly from informal sources such as friends, family members, or in-person or online dealers, are linked to most of the cases and play a major role in the outbreak (*1*,*7*,*8*). Thus, CDC and FDA recommend that persons not use tetrahydrocannabinol -containing e-cigarette, or vaping, products, particularly from informal sources. Vitamin E acetate, found in product samples tested by the FDA and state laboratories, has also been found in patient lung fluid specimens from a number of geographically diverse states tested by CDC (*9*). However, evidence is not yet sufficient to rule out the contribution of other chemicals of concern. While it appears that vitamin E acetate is associated with EVALI, there are many different substances and product sources that are being investigated, and there may be more than one cause. Therefore, the best way for persons to ensure that they are not at risk while the investigation continues is to consider refraining from the use of all e-cigarette, or vaping, products. Adults who continue to use e-cigarette, or vaping, products should carefully monitor themselves for symptoms and see a health care provider immediately if they develop symptoms similar to those reported in this outbreak (*5*,*10*). Irrespective of the ongoing investigation, e-cigarette, or vaping, products should never be used by youths, young adults, or pregnant women. Adults using e-cigarette, or vaping, products as an alternative to cigarettes should not go back to smoking; they should weigh all available information and consider using FDA-approved cessation medications.^§^

**Summary**

**What is already known about this topic?**

Some patients hospitalized for e-cigarette, or vaping, product use–associated lung injury (EVALI) have been rehospitalized or have died after hospital discharge.

**What is added by this report?**

Compared with other EVALI patients, rehospitalized patients and patients who died after hospital discharge were more likely to have one or more chronic conditions, including cardiac disease, chronic pulmonary disease, and diabetes, and to be older. At least one quarter of rehospitalizations and deaths occurred within 2 days after discharge.

**What are the implications for public health practice?**

Intensive discharge planning, ensuring clinical stability before discharge, optimized case management, and follow-up optimally within 48 hours after hospital discharge might minimize EVALI patients’ risk for rehospitalization and death, especially among patients with chronic conditions.

## Footnotes

*
https://www.cdc.gov/tobacco/basic_information/e-cigarettes/assets/2019-Lung-Injury-Surveillance-Case-Definition-508.pdf .

†
https://www.cdc.gov/tobacco/basic_information/e-cigarettes/severe-lung-disease/healthcare-providers/pdfs/National-Case-Report-Form-v01.pdf .

§ https://www.cdc.gov/tobacco/campaign/tips/quit-smoking/index.html?s_cid.
